# A cohort study investigating the relationship between patient reported outcome measures and pre-operative frailty in patients with operable, non-palliative colorectal cancer

**DOI:** 10.1186/s12877-020-01715-4

**Published:** 2020-08-27

**Authors:** J. Knight, K. Ayyash, K. Colling, J. Dhesi, V. Ewan, G. Danjoux, E. Kothmann, A. Mill, S. Taylor, D. Yates, Reema Ayyash

**Affiliations:** 1 Northern School of Anaesthesia and Intensive Care Medicine, South Tees Hospitals NHS Foundation Trust, Marton Road, Middlesbrough, TS4 3BW UK; 2 Department of Anaesthesia, York Teaching Hospital NHS Foundation Trust, Wigginton Road, York, YO31 8HE UK; 3 Department of Anaesthesia, South Tees Hospitals NHS Foundation Trust, Marton Road, Middlesbrough, TS4 3BW UK; 4 Department of Health and Ageing, Guy’s and St Thomas’ NHS Foundation Trust, London, SE1 7EH UK; 5 Department of Geriatric Medicine, South Tees Hospitals NHS Foundation Trust, Marton Road, Middlesbrough, TS4 3BW UK; 6 Department of Anaesthesia, North Tees and Hartlepool Hospitals NHS Foundation Trust, Hardwick Road, Hardwick, Stockton-On-Tees, TS19 8PE UK; 7 School of Natural and Environmental Sciences, University of Newcastle, Claremont Road, Newcastle upon Tyne, NE1 7RU UK

**Keywords:** Frailty, Edmonton frail scale, Clinical frailty scale, Colorectal cancer, Postoperative period, Patient reported outcome measures, Quality of life, EORTC QLQ-C30, WHO DAS

## Abstract

**Background:**

Frailty refers to the reduction in homeostatic reserve resulting from an accumulation of physiological deficits over a lifetime. Frailty is common in older patients undergoing surgery and is an independent risk factor for post-operative mortality, morbidity and increased length of hospital stay. In frail individuals, stressors, such as surgery, can precipitate an acute deterioration in health, manifesting as delirium, falls, reduction in mobility or continence, rendering these individuals at an increased risk of adverse perioperative outcomes. However, little is known about how frailty affects the patient experience, functional ability and quality of life (QoL) after surgery. In addition, the distribution of frailty in this population is unknown.

**Methods:**

We will conduct a multi-centre observational trial to investigate the relationship between patient reported outcome measures and preoperative frailty. We aim to recruit approximately two-hundred patients with operable, potentially curative colorectal cancer. Eligible patients will be identified at three hospital sites. QoL and functional ability (measured using EORTC QLQ-C30 and WHO-DAS 2.0 respectively) will be recorded at the pre-operative assessment clinic, and at 6 and 12 weeks postoperatively. Frailty scores including the Edmonton Frail Scale (EFS) and Rockwood clinical frailty scale (CFS) will be calculated both preoperatively, and at 12 weeks post-operatively. Secondary outcome measures including post-operative morbidity and mortality will be measured using Clavien Dindo classification and 90-day mortality.

**Discussion:**

This observational feasibility study seeks to define the prevalence of frailty in older (> 65 years) colorectal cancer patients and understand how frailty impacts on patient reported outcome measures. This information will help to inform larger studies relating to treatment decision algorithms and promote shared decision making in this population.

## Background

Frailty is a reduction in homeostatic reserve as a result of accumulated deficits through life, rendering affected individuals at increased risk of acute health deteriorations with stressor events e.g. surgery [1–3]. As death rates from cardiovascular disease have decreased significantly, the last 20 years has seen a significant expansion in the number of older people in the Western world. A consequence of this has been an increase in the number of people over the age of 75 years undergoing surgery; in England 1.5 million patients underwent surgery in 2006–7 [4] which increased to 2.5 million in 2014–2015 [5]. Of this latter group, 30% were > 85 years, and the prevalence of frailty in this age group is estimated to be 25–50% [2]. Surgical teams are operating on groups of patients that would previously have died before reaching the point of developing (for example) cancer.

There is reason to believe that the experience of surgery and its sequelae may be different in frailer patients, but at present there is little information to guide decision making for patients, surgeons and the surgical team. Patient reported outcome measures (PROMS) are a method of determining benefits of a particular procedure to patient quality of life (QoL). The association between frailty and PROMs: QoL and functional ability in the post-operative period are not clearly understood. Therefore, we seek to investigate the impact of frailty on PROMS in a group of patients with operable, non-palliative colorectal cancer.

Colorectal cancer is the 4th most common cancer in the UK [6], and is the 2nd commonest cause of cancer death in the UK, accounting for 10% of deaths [7]. The incidence of colorectal cancer is strongly related to age and is largely diagnosed in the elderly [8], when co-morbidities and frailty are common. Furthermore, evidence from a recent systematic review and meta-analysis has implicated frailty and co-morbidities to be strong prognostic factors of survival in colorectal cancer patients [9].

Moreover, studies to date have demonstrated that older patients often prioritise treatment outcomes - functional independence and cognition, more than survival [10, 11]. Ronning et al. (2016), demonstrated in a follow-up study of patients who underwent surgery for colorectal cancer, a clinically significant improvement in emotional functioning in the sub-group of frail patients when compared to their non-frail counterparts, however, no significant change in physical functioning was observed. Interestingly, although improvements in QoL-scores were demonstrated in the total cohort, but to lesser extent in frail individuals, the trajectory of scores were similar to the non-frail group [12].

Understanding how pre-operative frailty affects PROMs may facilitate and support better collaborative decision-making by providing patients with reliable information on what to expect from cancer surgery [13–15]. In addition, it may provide clinicians with a better understanding of the trajectories of frail versus non-frail individuals following surgery, thus allowing for tailored perioperative optimisation of individuals and permitting modifications to standard treatments that would otherwise render individuals of high-risk complications.

We aim to test the hypothesis that preoperative frailty in older patients undergoing surgery for operable non-palliative colorectal cancer is positively associated with post-operative functional ability and inversely associated with postoperative QoL. We also aim to understand the distribution of frailty in this population.

## Methods/design

### Study design and setting

This is a multi-centre prospective observational feasibility study. The study will be conducted across three sites – South Tees NHS Foundation Trust (STHNFT), University Hospital of North Tees (UHNT) and York Teaching Hospital NHS Foundation Trust (YTHNFT).

### Patient population and sample characteristics

#### Inclusion criteria

- Colorectal cancer diagnosis (as defined by NICE 2016) with potentially curable disease at radiological staging
- Age ≥ 65 years
- Patients who lack ability to consent but have a personal consultee who agrees to sign the ‘Consultee Declaration Form’

#### Exclusion criteria

- Age < 65 years
- Day case procedure
- Receiving palliative surgery for colorectal cancer diagnosis
- Do not understand and speak English
- Do not have cancer but undergoing major bowel surgery
- Patients who do not provide written informed consent and whose personal consultee declines to sign the ‘Consultee Declaration Form”.

### Recruitment

The SPIRIT timeline (Table 1) shows the research project projected timeframe. All consecutive patients with operable, non-palliative colorectal cancer will be identified by the colorectal specialist nurses in the surgical outpatient clinics and informed about the study. Patients who express an interest in the study will be referred to the research team. This may include a minority of patients who are subsequently deemed unfit for surgery, who will be analysed as a subgroup. Invitation letters and a participant information leaflet will be sent out to patients who have expressed an interest in the study prior to the preoperative assessment clinic appointment. Patients will be assessed for capacity to consent using clinical judgment and standard procedures. Written consent will be obtained in the pre-assessment clinic via the designated consent form for patients wishing to participate by a study investigator. In patients who lack capacity to consent, a personal consultee will be counselled and asked to sign the declaration form. The personal consultee will be identified by the research team in accordance with the Department of Health Guidance on nominating a consultee for research involving adults who lack capacity to consent – Section 32(3) of the Mental Capacity Act 2005. The personal consultee is defined as an individual who knows the person who lacks capacity well but is not acting in a professional or paid capacity. In the absence of a personal consultee, a nominated consultee will not be sought. This process will be consistent across sites.

**Table 1:** SPIRIT recruitment schedule

	STUDY PERIOD						
	Preparation	Enrolment	Post-Surgery Phase				Completion
*Timepoint	-t_**1**_	t_**0**_	t_**1**_	t_**2**_	t_**3**_	t_**4**_	t_**x**_
**Preparation Phase**							
REC Approval	X						
HRA Approval	X						
R&D Approval	X						
Site initiation visit	X						
Statistical plan write up	X						
Collection proforma	X						
Submit work to sites	X						
**Enrolment Phase**							
Eligibility screening		X					
Informed consent		X					
Recruitment		X					
Baseline variables		X					
**Post-Surgery Phase**							
Outcome variables			X	X			
Other data variables			X	X			
**Project Completion**							
Data analysis							X
Statistical write-up							X
Close Study							X
Dissemination of results							X
Other Milestones							
Patient Focus Groups					X	X	
Reports to NIAA					X		X

*Timepoint = -t_1_= project preparation, t_0_ = baseline, t_1_ = 6 weeks, t_2_ = 12 weeks, t_3_ = 9–12 months, t_4_ = 17–20 months

### Sample size estimation

**Fig. 1:**
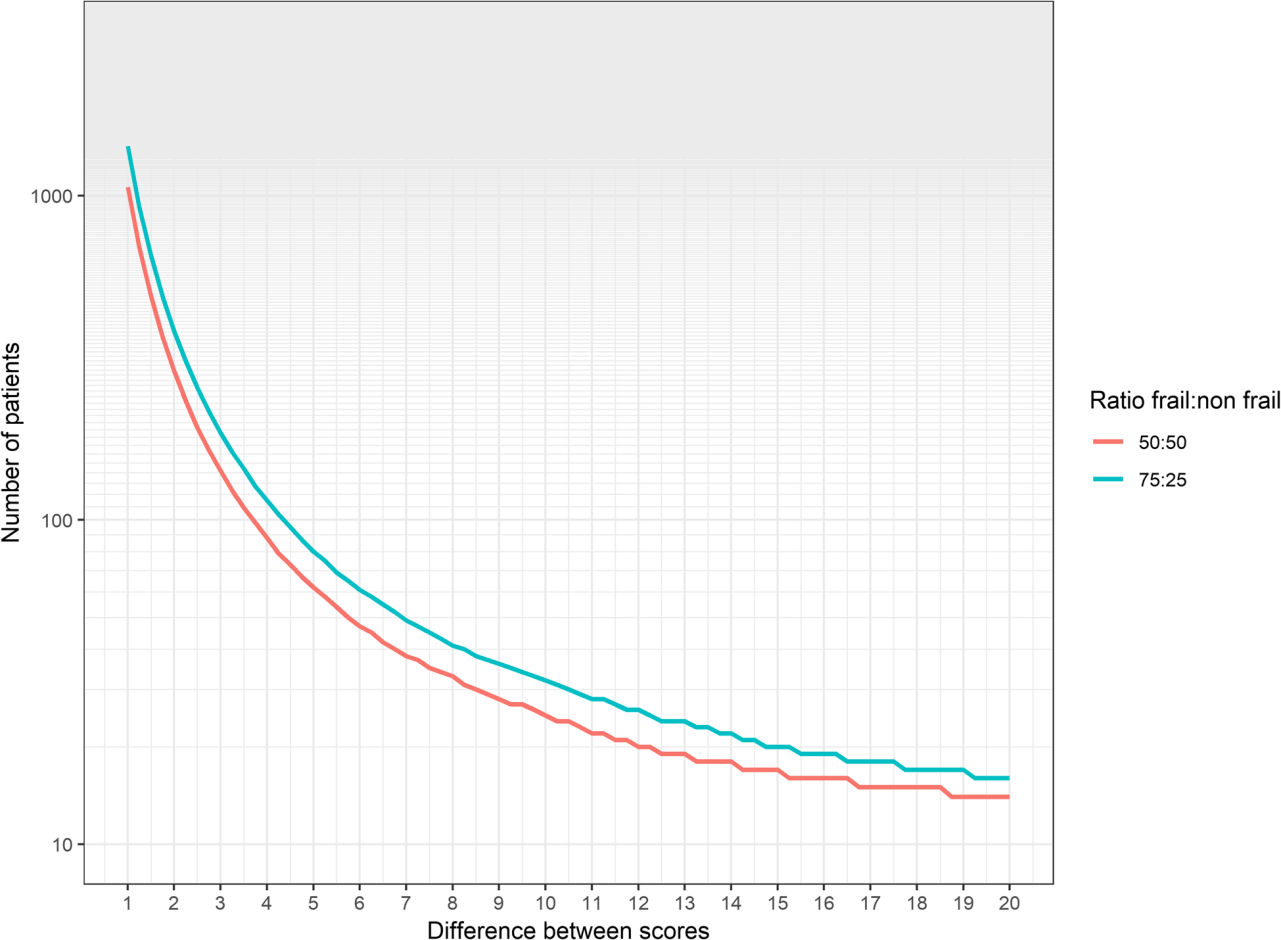
Power analysis to determine sample size required to detect difference in PROM scores

Using current data, we estimate that over an 18-month recruitment period across the three sites, approximately 817 cancer resections will be performed. A realistic recruitment target is thought to be 25% across these sites. We anticipate recruitment of frail and non-frail patients to be in line with that observed at other centres with approximately 25% of patients presenting as frail. We estimate the difference in PROM scores between frail patients and non-frail patients would be approximately 6 points for the EORTC QLQ-C30 questionnaire and 12 points on the WHO-DAS [16]. A sample size of 200 patients would comfortably allow us to detect this level of difference between frail and non-frail persons at 80% power and the 0.05 significance level (Fig. 1). There is no previous work to guide this estimate, so we will review our power calculation after 6 months of recruitment.

## Cognition, frailty, quality of life and functional ability measures

### Cognition

Assessment of cognitive impairment will be performed using the Montreal Cognitive Assessment Tool (MoCA) [17]. This is a brief screening tool for the detection of mild cognitive impairment. It is a one-page 30 point test that takes approximately 10 min to complete. We chose this tool as it is quick to administer and assesses several cognitive domains; memory recall, visuospatial abilities, language, orientation to time and place, attention/concentration and working memory and multiple aspects of executive function.

### Frailty

Assessment of frailty will be performed using the Edmonton Frail Scale (EFS) (Additional File One) [18, 19] and Clinical Frailty Scale (CFS) (Additional File Two) [20]. We chose these tools for assessment of frailty as these are simple, validated, time-efficient tools that work best in the preoperative setting for screening of frailty [21, 22]. A score of ≥8 on the EFS and a score ≥ 5 on CFS indicate the presence of frailty in an individual. We will collect the score for both assessment tools to determine which tool is more pragmatic to use in the preoperative setting. Additionally, we will investigate the relationship between EFS and CFS scores and PROM scores to determine whether one frailty score correlates more strongly with patient related outcomes in the elective setting.

### Primary outcome measures

Our PROM questionnaires were informed by a literature review and discussion with the stakeholder group including a patient focus group. The questionnaires decided upon are the European Organization for Research and Treatment of Cancer Quality of Life Questionnaire-Core 30 item (EORTC QLQ-C30) (Additional File Three) [23], and the World Health Organisation Disability Assessment Schedule (WHO-DAS 12 item version 2.0) (Additional File Four) [24].

The EORTC QLQ-C30 is a reliable, validated patient reported outcome questionnaire for measuring cancer-specific health-related QoL. The questionnaire is user-friendly and takes approximately 11 min to complete unaided [25]. WHO Disability Assessment Scale 2.0 (WHO-DAS II) is a validated tool for collecting data on social, physical and occupational impairments in the general population and patient specific groups which have occurred due to ill-health. It can be self-administered and takes up to 5 min to complete [26]. The EORTC QLQ-C30 and WHO-DAS 2.0 can be completed by a third party such as a family member or care giver in patients with cognitive impairment [24, 27].

### Secondary outcome measures

Clavien Dindo Classification system is a widely-accepted categorisation system for post-operative complications into four groups, graded according to severity [28].

The secondary outcome measures will be collected to determine the association between preoperative frailty and:

- Postoperative morbidity using the Clavien Dindo classification. This will be collected on days 1,3,5 and 8 post-surgery
- 90-day mortality
- Days alive and at home 30 days post operatively (DAH 30)

### Data collection

**Fig. 2:**
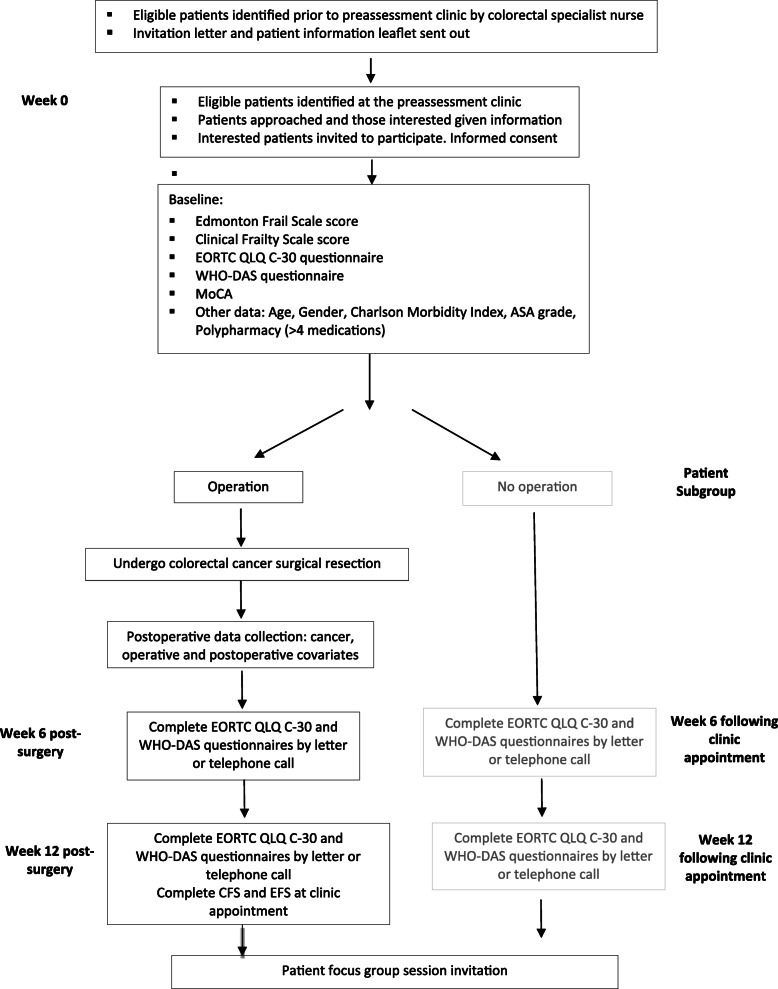
Patient flow and data collection

The data collection is shown in the flowchart Fig. 2. Following recruitment, a researcher will complete the EFS and CFS at the pre-assessment clinic and support participants (or their carers) to complete the EORTC QLQ C30 and WHO-DAS II questionnaires.

After surgery, all participants will be invited to repeat the PROM questionnaires via a telephone call or letter approximately 6 and 12-weeks postoperatively in agreement with the patient or their carers preferred method. Frailty assessments will also be repeated approximately 12 weeks during routine surgical follow-up. In patients who do not undergo surgery, PROM questionnaires will be completed approximately 6 and 12-weeks after the preoperative clinic.

In patients who have cognitive impairment in accordance with the Montreal Cognitive Assessment score ≤ 26 [29], and who are unable to complete the assessment tools, these will be completed by the patient representative/next of kin. 

In addition, the researchers will collect preoperative and postoperative data via prospective case notes review as per Table 2.

**Table 2:** Covariates and outcome measures to be collected

Category of covariate	Data to be collected
**Patient covariates**	Age
	Height
	Sex
	Weight
	Preoperative Charlson Comorbidity Index
	ASA grade
	Polypharmacy (> 4 medications)
	CFS
	EFS
	Cognitive impairment using Montreal Cognitive Assessment (MoCA)
**Cancer covariates**	TNM stage
	Tumour location
	Preoperative chemo/radiotherapy
**Operative covariates**	Procedure or not
	Procedure type
	Duration of surgery
**Postoperative covariates**	Postoperative chemo/radiotherapy
	Major postoperative complications
**Primary Outcome measures**	EORTC QLQ C30 at 6 and 12 weeks
	WHO-DAS 12 item at 6 and 12 weeks
	CFS and EFS at 12 weeks
**Secondary outcome measures**	Clavien Dindo (Postoperative morbidity) – collected on days 1,3,5 and 8 post-surgery
	90-day Mortality
	Days alive and at home 30-days (DAH30)

The data will be collected by trained research nurses or the investigators. The study will be conducted in accordance with Good Clinical Practice. Data management and analysis will be complied in line with General Data Protection Regulations and the Data Protection Act 2018.

### Data analysis

Data will be anonymised, stored and cleaned in an Excel spreadsheet held on the Trust server. Consent will be obtained to transfer the anonymised dataset to Newcastle University for analysis. Data will be analysed using R [30]. WHO-DAS II will be scored using Item Response Theory, and the EORTC QLQ C30 will be scored using linear transformation. Mixed effects modelling using ordinal logistic regression (clmm package) will be used to investigate how PROMs are associated with EFS and CFS. The Vegan package will be used to correlation between the scores via an ordination approach.

In order to minimise potential sources of bias during participant recruitment, all researchers will be trained in using the frailty scores and calibrated using hypothetical cases before the start of study to reduce information bias. Researchers will also receive training on conducting the MoCA tool. Researchers will meet after recruiting the first 10 patients to discuss and resolve any questions about interpretation of assessment tools. We will describe inter-observer variability in frailty scoring with both scores and report this as part of our findings. We aim to reduce bias during PROM follow-up interviews (face to face and telephone) by conducting the interview using the same 4 trained researchers.

We will analyse the distribution of frailty scores (Rockwood scores collected pre-operatively in all patients, regardless of whether recruited into the study) of patients who choose not to enter the study in order to identify any potential selection bias.

## Discussion

Colorectal cancer is prevalent in the older population, when comorbidities and frailty are common, with an average of more than 4 in 10 (44%) new cases of colorectal cancer diagnosed each year in people aged 75 years and over [8]. Chronological age alone is a poor predictor of cancer treatment tolerance. In addition, several studies have identified frailty as an independent predictor of survival in older patients with colorectal cancer [9, 31]. Preoperative assessment of frailty is not current standard practice despite compelling evidence that it is common in older patients and is associated with increased post-operative morbidity and mortality [32–38]. Failure to detect frailty potentially exposes older cancer patients to treatments that might not benefit them and undeniably harm them. Therefore, the heterogeneity of the older cancer patient necessitates a judiciously tailored approach to the surgical and anaesthetic care of the individual patient that considers frailty.

Evidence from several studies have implicated that older patients frequently prioritise functional outcomes following surgery over survival, and this may be true for the older, frail patient with limited life expectancy. Despite this, there is a paucity of literature investigating the impact of frailty on patient reported outcome measures, mainly quality of life and functional ability.

The information obtained from this prospective observational feasibility study is pivotal for facilitating and supporting better collaborative decision-making by providing patients with reliable information on what to expect from cancer surgery based on an individualised balance of risk and benefit. In addition, it will provide healthcare professionals with a better understanding of the trajectories of frail versus non-frail following surgery, thus providing an opportunity for tailored perioperative optimisation of individuals and permitting modifications to standard treatments that would otherwise render individuals of high risk complications.

The prevalence of frailty in colorectal cancer patients in the UK is unknown. Better knowledge of the epidemiology of frailty in older colorectal cancer patients is essential to improving cancer care of this older patient population group. It is expected that the majority of patients presenting to the surgical outpatient clinic with operable colorectal cancers will be non-frail. This may be due to the non-frail patients being more likely to engage with screening programmes, being more vigilant for red flag symptoms for cancer or being more likely to be referred for investigation. Although we are more likely to obtain information pertaining to non-frail patients, it will better inform the prevalence of this patient sub-group in real life setting. The analysis framework does not require a balanced number of frail and non-frail patients but any imbalance should be minimised during recruitment. The mixed model framework allows interpretation of the data collected while accounting for any bias associated with the full range of covariates.

Patients will be invited to attend a focus group at 2 intervals - midpoint (9–12 months) and prior to project completion (17–20 months). The midpoint feedback will identify any interim problems that require addressing such as barriers to recruitment or completion of questionnaires. The end of project feedback will provide information regarding patients’ experience of participating in the study, help inform dissemination strategy of information to future participants on study completion for example via a newsletter and aid with planning the intervention or future studies. The study results will be disseminated to professionals via local directorate presentation, conference presentation and publication.

To conclude, we will describe the distribution of frailty in a colorectal cancer population and investigate the relationship between preoperative frailty and post-operative patient related outcome measures. A better understanding of this subject will support routine assessment of frailty in the older cancer patient presenting for surgery as well as provide patients and healthcare professionals with reliable information pertaining to health outcomes following surgery that may help facilitate healthcare providers and recipient’s decision and policy making process. It will also aid the planning of service provision and guide the development of future elective surgical pathways.

## Supplementary information

**Additional file 1.** Edmonton Frail Scale.

**Additional file 2.** Clinical Frailty Scale.

**Additional file 3.** European Organization for Research and Treatment of Cancer Quality of Life Questionnaire-Core 30 item.

**Additional file 4.** World Health Organisation Disability Assessment Schedule (WHO-DAS 12 item version 2.0).

## Data Availability

Not applicable.

## Abbreviations

PROMs: Patient Reported Outcome Measures
QoL: Quality of Life
STHNFT: South Tees Hospital NHS Foundation Trust
UHNT: University Hospital North Tees
YTHNFT: York Teaching Hospital NHS Foundation Trust
EFS: Edmonton Frail Scale
CFS: Clinical Frailty Scale
EORTC QLQ-C30: European Organisation for Research and Treatment of Cancer Quality of Life Questionnaire- Core 30 item
WHO-DAS: World Health Organisation Disability Assessment Schedule

## References

[1] Beggs T, Sepehri A, Szwajcer A, Tangri N, Arora RC (2015). Frailty and perioperative outcomes: a narrative review. Can J Anesth.

[2] Clegg A, Young J, Iliffe S, Rikkert MO, Rockwood K (2013). Frailty in elderly people. Lancet.

[3] Xue Q-L (2011). The frailty syndrome: definition and natural history. Clin Geriatr Med.

[4] Health and social care information centre – hospital episode statistics, admitted patient care – England, 2006-2007: Main operations summary. https://digital.nhs.uk/data-and-information/publications/statistical/hospital-admitted-patient-care-activity/hospital-episode-statistics-admitted-patient-careengland-2006-07. Accessed 7 May 2019.

[5] Health and social care information centre – hospital episode statistics, admitted patient care – England, 2014-2015: Procedures and interventions. https://digital.nhs.uk/data-and-information/publications/statistical/hospital-admitted-patient-care-activity/hospital-episode-statistics-admitted-patient-careengland-2014-15. Accessed 7 May 2019.

[6] Cancer Research UK - Bowl Cancer Incidence Statisitics. https://www.cancerresearchuk.org/health-professional/cancer-statistics/statistics-by-cancer-type/bowel-cancer/incidence#heading-Zero.

[7] Cancer Research UK - Bowl Cancer Motality by Sex and UK Country. https://www.cancerresearchuk.org/health-professional/cancer-statistics/statistics-by-cancer-type/bowel-cancer/mortality#heading-Zero.

[8] Cancer Research UK – Bowl Cancer Incidnece by Age. https://www.cancerresearchuk.org/health-professional/cancer-statistics/statistics-by-cancer-type/bowel-cancer/incidence#heading-One.

[9] Boakye D, Rillmann B, Walter V (2018). Impact of comorbidity and frailty on prognosis in colorectal cancer patients: a systematic review and meta-analysis. Cancer Treat Rev.

[10] Akishita M, Ishii S, Kojima T (2013). Priorities of health care outcomes for the elderly. J Am Med Dir Assoc.

[11] Fried TR, Bradley EH, Towle VR, Allore H (2002). Understanding the treatment preferences of seriously ill patients. N Engl J Med.

[12] Ronning B, Wyller TB, Nesbakken A (2016). Quality of life in older and frail patients after surgery for colorectal cancer—a follow-up study. J Geriatr Oncol.

[13] Bougeard A, Snowden C, Swart M (2017). Shared Decision Making in Practice Part I.

[14] Bougeard A, Snowden C, Swart M (2017). Shared decision making in practice. Part II.

[15] Bougeard A, Snowden C, Swart M (2018). Shared decision making in practice. Part III.

[16] Cocks K, King MT, Velikova G, Martyn St-James M, Fayers PM, Brown JM (2011). Evidence-based guidelines for determination of sample size and interpretation of the European organisation for the research and treatment of Cancer quality of life questionnaire Core 30. J Clin Oncol.

[17] Montreal Cognitive Assessment Tool. https://www.mocatest.org/.

[18] Edmonton Frailty Scale. https://www.cgakit.com/fr-1-edmonton-frail-scale.

[19] Meyers BM, Al-Shamsi HO, Rask S (2017). Utility of the Edmonton frail scale in identifying frail elderly patients during treatment of colorectal cancer. J Gastrointest Oncol.

[20] Rockwood Clinical Frailty Scale. https://www.cgakit.com/fr-1-rockwood-clinical-frailty-scale.

[21] Rockwood K, Song X, MacKnight C (2005). A global clinical measure of fitness and frailty in elderly people. CMAJ.

[22] Van Kan A, Rolland Y, Bergman H (2008). The I.A.N.A Task Force on frailty assessment of older people in clinical practice. J Nutr Health Aging.

[23] EORTC QLQ-C30 https://qol.eortc.org/questionnaire/eortc-qlq-c30/.

[24] WHO. Disability Assessment Schedule 2.0. https://www.who.int/classifications/icf/whodasii/en/index3.html.

[25] Aaronson NK, Aherdzai S, Bergman B, Bullinger M, Cull A (1993). The European Organisation for Research and Treatment of Cancer QLQ-C30: a quality of life instrument for the use in international clinical trials in oncology. J Natl Cancer Inst.

[26] Measuring health and disability. Manual for WHO disability assessment schedule (WHODAS2.0). http://apps.who.int/iris/bitstream/handle/10665/43974/9789241547598_eng.pdf;jsessionid=E46E7B68C15E675FD466B3B32FC6BB0A?sequence=1.

[27] QoL Clinical Trials Guidelines. https://www.eortc.org/app/uploads/sites/2/2018/02/clinical_trials__guidelines_qol.pdf.

[28] Dindo D, Demartines N, Clavien P-A (2004). Classification of surgical complications: a new proposal with evaluation in a cohort of 6336 patients and results of a survey. Ann Surg.

[29] Milani SA, Marsiske M, Cottler LB (2018). Optimal cutoffs for the Montreal cognitive Assessment vary by race and ethnicity. Alzheimers Dement (Amst).

[30] R Core Team (2013). R: a language and environment for statistical computing.

[31] Ommundsen N, Wyller TB, Nesbakken A (2014). Frailty is an independent predictor of survival in older patients with colorectal cancer. Oncologist.

[32] Beech F, Brown A, Dhesi J, Foo I, Goodall J, Membership of the working party RG (2014). Peri-operative care of the elderly. Anaesthesia.

[33] Aihie Sayer A, Cooper C, Gale CR (2014). Prevalence of frailty and disability: findings from the English longitudinal study of ageing. Age Ageing.

[34] Lin H-S, Watts JN, Peel NM, Hubbard RE (2016). Frailty and post-operative outcomes in older surgical patients: a systematic review. BMC Geriatr.

[35] Ambler GK, Brooks DE, Al Zuhir N, Ali A, Gohel MS, Hayes PD (2015). Effect of frailty on short- and mid-term outcomes in vascular surgical patients. BJS..

[36] Hamel MB, Henderson WG, Khuri SF, Daley J (2005). Surgical outcomes for patients aged 80 and older: morbidity and mortality from major noncardiac surgery. J Am Geriatr Soc.

[37] Robinson TN, Wu DS, Pointer L, Dunn CL, Cleveland JC, Moss M (2013). Simple frailty score predicts postoperative complications across surgical specialties. Am J Surg.

[38] Dasgupta M, Rolfson DB, Stolee P, Borrie MJ, Speechley M (2009). Frailty is associated with postoperative complications in older adults with medical problems. Arch Gerontol Geriatr.

