# Supplementary material for: A cohort study investigating the relationship between patient reported outcome measures and pre-operative frailty in patients with operable, non-palliative colorectal cancer
Source: BMC Geriatr. 2020 Aug 27;20:311. doi: 10.1186/s12877-020-01715-4 (PMC7453711; doi:10.1186/s12877-020-01715-4)
Supplement: Supplementary file 1 — Additional file 1. Edmonton Frail Scale. [file 12877_2020_1715_MOESM1_ESM.docx]

**Additional File One: Edmonton Frail Scale**

| **Frailty domain** | **Item** | **0** | **1** | **2** |
| --- | --- | --- | --- | --- |
| **Cognition** | Please imagine that this pre-drawn circle is a clock. I would like you to place the numbers in the correct positions then place the hands to indicate a time of ‘ten after eleven’ | No errors | Minor spacing errors | Other errors |
| **General health status** | In the past year how many times have you been admitted to the hospital? | 0 | 1–2 | ≥2 |
|  | In general, how do you describe your health? | Excellent’, ‘Very good’, ‘Good’ | ‘Fair’ | ‘Poor’ |
| **Functional independence** | With how many of the following activities do you require help?(meal preparation, shopping, transportation, telephone, housekeeping, laundry, managing money, taking medications) | 0–1 | 2–4 | 5-8 |
| **Social support** | When you need help, can you count on someone who is willing and able to meet your needs? | Always | Sometimes | Never |
| **Medication use** | Do you use five or more different prescription medications on a regular basis? | No | Yes |  |
|  | At times, do you forget to take your prescription medications? | No | Yes |  |
| **Nutrition** | Have you recently lost weight such that your clothing has become looser? | No | Yes |  |
| **Mood** | Do you often feel sad or depressed? | No | Yes |  |
| **Continence** | Do you have a problem with losing control of urine when you don’t want to? | No | Yes |  |
| **Functional performance** | I would like you to sit in this chair with your back and arms resting. Then, when I say ‘GO’, please stand up and walk at a safe and comfortable pace to the mark on the floor (approximately 3 m away), return to the chair and sit down’ | 0–10 s | 11–20 s | One of : >20s,or patient unwilling , or requires assistance |
| **Totals** | Final score is the sum of column totals |  |  |  |

**Scoring: Total Score**_____/17

0-5 = Not Frail

6-7 = Vulnerable

8-9 = Mild Frailty

10-11 = Moderate frailty

12-17 = Severe Frailty
