# Supplementary figures and images for: A cohort study investigating the relationship between patient reported outcome measures and pre-operative frailty in patients with operable, non-palliative colorectal cancer
Source: BMC Geriatr. 2020 Aug 27;20:311. doi: 10.1186/s12877-020-01715-4 (PMC7453711; doi:10.1186/s12877-020-01715-4)

**Additional File Two: Clinical Frailty Scale**


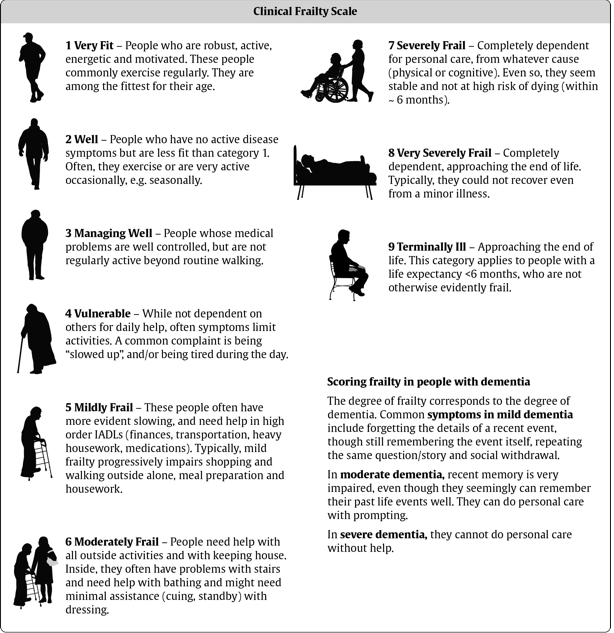

Supplement: Supplementary file 2 — Additional file 2. Clinical Frailty Scale. [file 12877_2020_1715_MOESM2_ESM.docx]
