# Supplementary material for: A cohort study investigating the relationship between patient reported outcome measures and pre-operative frailty in patients with operable, non-palliative colorectal cancer
Source: BMC Geriatr. 2020 Aug 27;20:311. doi: 10.1186/s12877-020-01715-4 (PMC7453711; doi:10.1186/s12877-020-01715-4)

**Additional File Three: European Organization for Research and Treatment of Cancer Quality of Life Questionnaire-Core 30 item**


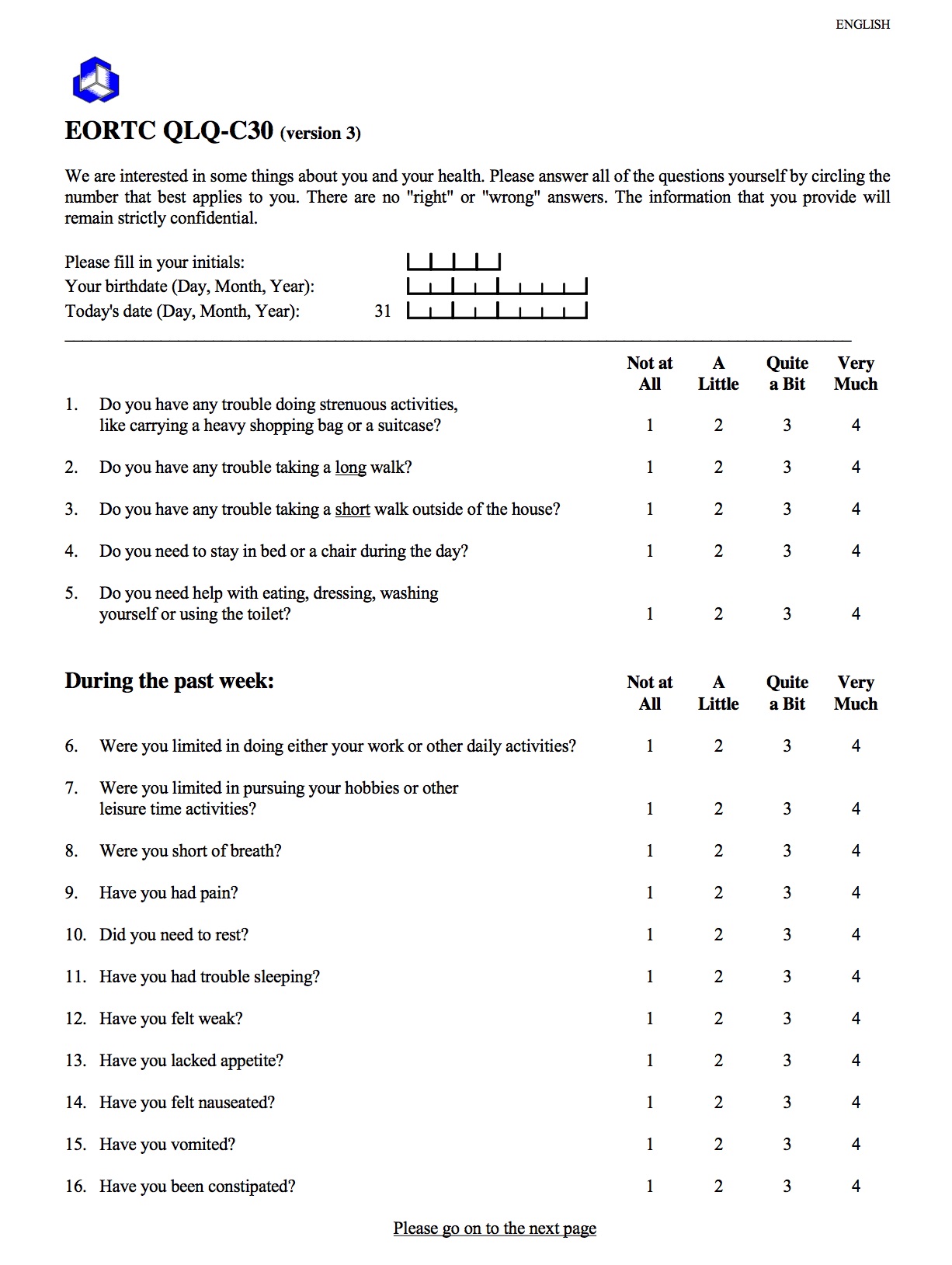


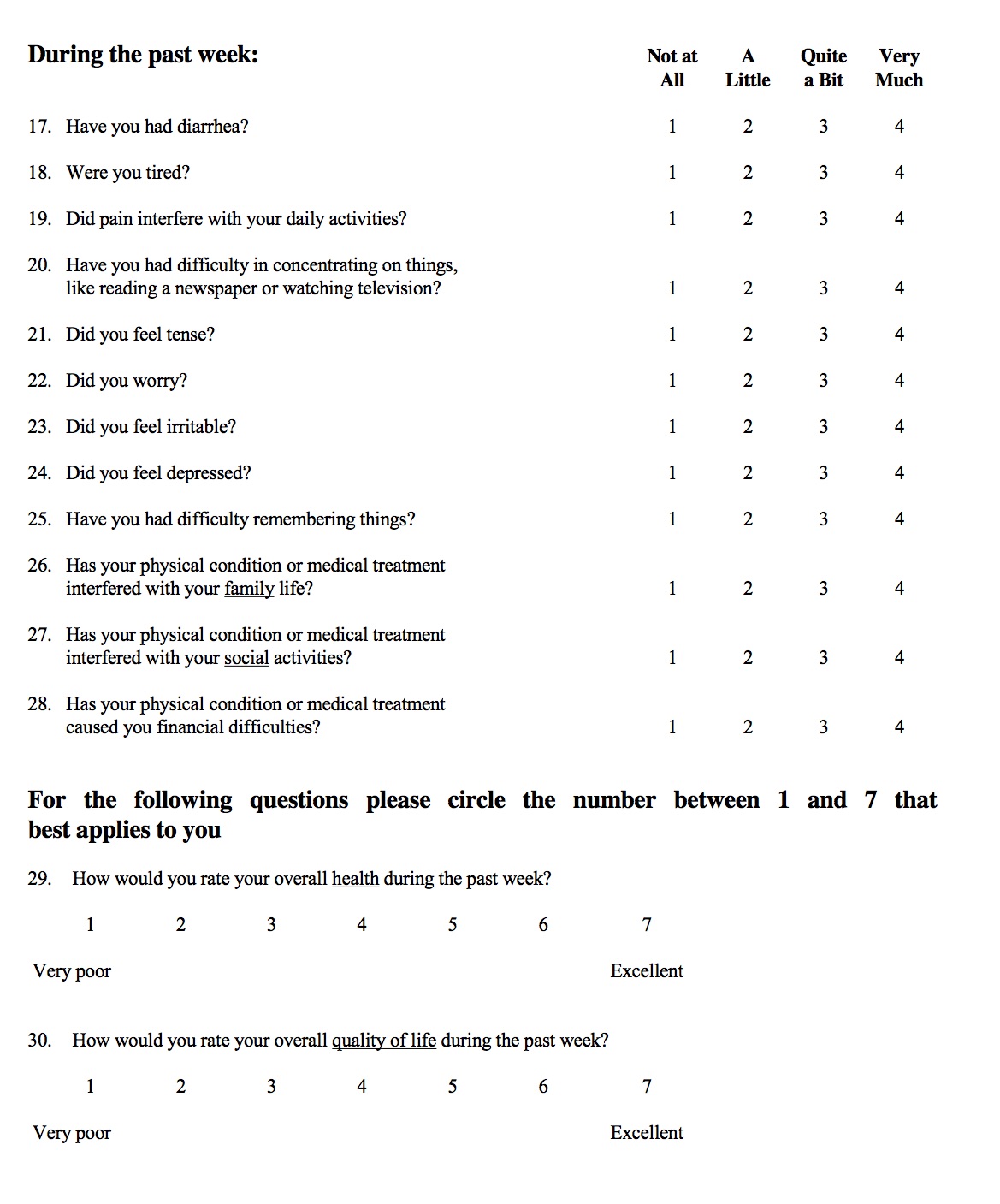

Supplement: Supplementary file 3 — Additional file 3. European Organization for Research and Treatment of Cancer Quality of Life Questionnaire-Core 30 item. [file 12877_2020_1715_MOESM3_ESM.docx]
