# Supplementary material for: A cohort study investigating the relationship between patient reported outcome measures and pre-operative frailty in patients with operable, non-palliative colorectal cancer
Source: BMC Geriatr. 2020 Aug 27;20:311. doi: 10.1186/s12877-020-01715-4 (PMC7453711; doi:10.1186/s12877-020-01715-4)

**Additional File Four: World Health Organisation Disability Assessment Schedule (WHO-DAS 12 item version 2.0)**


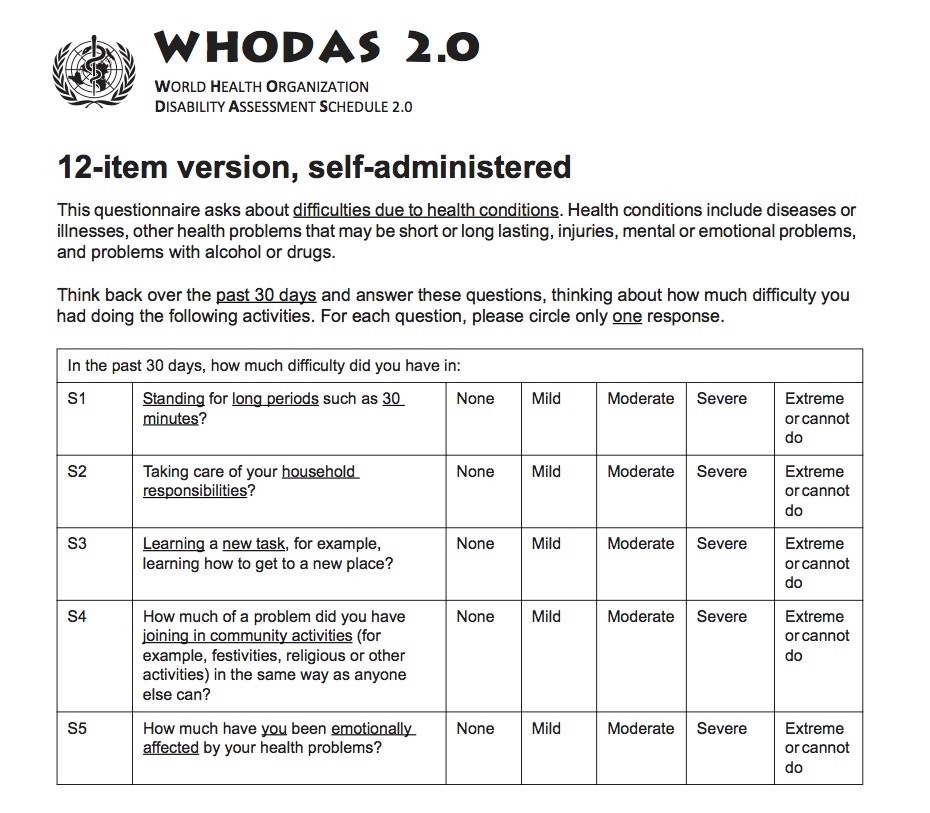


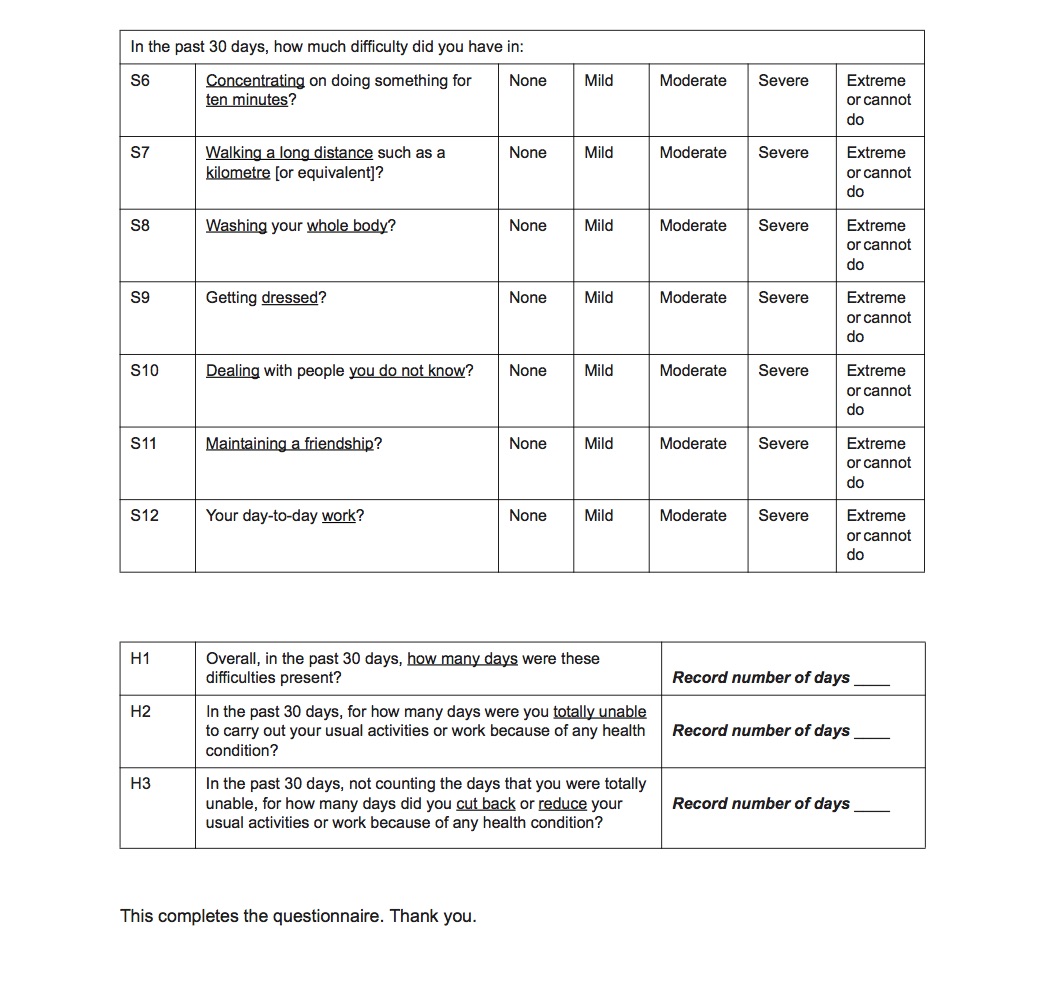

Supplement: Supplementary file 4 — Additional file 4. World Health Organisation Disability Assessment Schedule (WHO-DAS 12 item version 2.0). [file 12877_2020_1715_MOESM4_ESM.docx]
